# Cardiovascular Considerations in Coronavirus Disease 2019 with a Special Focus on Arrhythmia

**DOI:** 10.19102/icrm.2020.110804

**Published:** 2020-08-15

**Authors:** Tiffany Y. Hu, Justin Z. Lee, Samuel J. Asirvatham

**Affiliations:** ^1^Department of Cardiovascular Medicine, Mayo Clinic, Rochester, MN, USA; ^2^Division of Pediatric Cardiology, Department of Pediatric and Adolescent Medicine, Mayo Clinic, Rochester, MN, USA

**Keywords:** Arrhythmia, cardiovascular, COVID-19, SARS-CoV-2, myocarditis

## Abstract

Severe acute respiratory syndrome coronavirus 2 (SARS-CoV-2), the coronavirus responsible for the coronavirus disease 2019 (COVID-19) pandemic, has significant cardiovascular manifestations. Several studies to date have suggested worse outcomes occur in patients with elevated troponin levels. Among hospitalized patients in Wuhan, China, arrhythmias including malignant ventricular arrhythmia have been reported. Conduction abnormalities in COVID-19 patients have also been described. Additionally, there have been concerns raised regarding COVID-19–related myocarditis, of which reported biopsy-proven cases to date appear to be rare. In this review, we address COVID-19 concerns for the cardiologist and electrophysiologist, including arrhythmia and conduction abnormalities, myocarditis, and arrhythmia in critically ill patients; angiotensin-converting enzyme 2 in cardiac patients; hypercoagulability; and the drug properties of hydroxychloroquine as one of the potential therapies under review.

## Introduction

Coronavirus disease 2019 (COVID-19) is caused by severe acute respiratory syndrome coronavirus 2 (SARS-CoV-2), an enveloped, positive sense, single-stranded RNA virus.^1^ SARS-CoV-2 shares the same genus as the Middle East respiratory syndrome-related coronavirus and the same virus species as severe acute respiratory syndrome coronavirus (SARS-CoV).^1^ SARS-CoV-2 enters host cells by binding to functional receptor angiotensin-converting enzyme 2 (ACE2), an enzyme otherwise well-known for its role in regulating the renin–angiotensin system. ACE2 is widely expressed in the human body, including in the endothelium and smooth muscle of the vasculature and in organs such as the heart, kidneys, lungs, and gastrointestinal tract.^2,3^

Since the first report of SARS-CoV-2 originated from Wuhan, China in late 2019—with some reports differing on whether the index case was in November or December of that year—and the declaration of a pandemic by the World Health Organization in March 2020, there have been several studies conducted regarding the intersection of COVID-19 and cardiovascular disease. Observations of particular interest include the higher case fatality rate in patients with COVID-19 and cardiovascular disease, the association of troponin elevation with worse prognosis, the importance of ACE2 in COVID-19, and electrocardiogram (ECG) findings in reported cases of myocarditis.^4–7^

**Figure 1:**
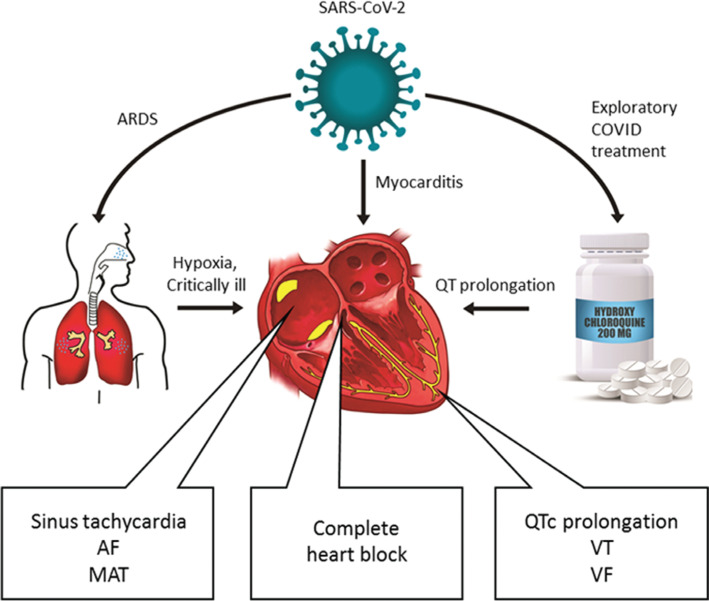
Rhythm disturbances in the setting of SARS-CoV-2 infection may result from (1) critical illness, specifically acute respiratory distress syndrome (ARDS); (2) myocarditis with associated ECG changes; (3) bradyarrhythmia and tachyarrhythmia; and (4) QTc prolongation and rare reports of ventricular arrhythmia as a side effect of the potential COVID-19 pharmacotherapy hydroxychloroquine.

In this review, we highlight a variety of findings reported to date including arrhythmias in patients with COVID-19, reported cases of myocarditis, ECG changes in an animal model infected with coronavirus, arrhythmias in critically ill patients, cardiotropic viruses and arrhythmia, implications of ACE2 in cardiovascular disease, hypercoagulability, and hydroxychloroquine for the electrophysiologist **(Figure 1)**.

## Arrhythmia in COVID-19

Underlying cardiovascular disease is associated with increased mortality in patients with COVID-19.^4,8^ However, cardiac complications of SARS-CoV-2 infection including troponin elevation, reduced systolic function, cardiogenic shock, myocarditis, and arrhythmia have been reported even in patients without pre-existing cardiovascular disease. Possible mechanisms of cardiac injury include cytokine storm, increased sympathetic tone, supply–demand mismatch, exacerbation of underlying disease, hypercoagulability, and direct cardiac involvement. Ultimately, until the mechanisms of cardiac injury associated with SARS-CoV-2 infection are more clearly elucidated, the mechanism of arrhythmia in the setting of COVID-19 remains speculative, although more data are anticipated to be forthcoming.

Two studies have described the incidence of arrhythmia in patients hospitalized with COVID-19 pneumonia in Wuhan, China. Wang et al. reported arrhythmias in 16% of hospitalized COVID-19 patients.^9^ These authors compared the characteristics of patients requiring intensive care unit (ICU) admission and those who did not, finding among critically ill patients that the rates of acute cardiac injury (defined as troponin elevation or new ECG or echocardiographic changes) and arrhythmia (44% versus 7%) were higher. In the study by Guo et al., ventricular tachycardia (VT) or ventricular fibrillation (VF) was reported in 6% of hospitalized COVID-19 patients.^5^ Patients with known cardiovascular disease and troponin T elevation were reported to experience the greatest rate of mortality. Information regarding patients’ baseline corrected QT interval (QTc), the use of QT-prolonging medications such as hydroxychloroquine, and/or the direct cardiac involvement of SARS-CoV-2 was not reported in these studies.

Shao et al. studied the etiology of in-house cardiopulmonary arrest in patients admitted to a dedicated COVID-19 hospital in Wuhan with severe COVID-19 pneumonia. Of 761 admitted patients, the outcomes of 136 resuscitated patients were analyzed.^10^ The initial rhythm during cardiopulmonary arrest was asystole in 90% of patients; pulseless electrical activity in 4%; and a shockable rhythm, defined as VF or pulseless VT, in 6%. The return of spontaneous circulation was achieved in 13% of patients and the 30-day survival rate was 3%. The majority of cardiopulmonary arrests were of a respiratory etiology (88%).

## Conduction disease in COVID-19

To date, there have been rare case reports describing the development of transient heart block in critically ill COVID-19 patients.^11,12^ In professional society online discussions, first-, second-, and third-degree heart block have also been reported anecdotally.^13,14^ Azarkish et al. introduced the case of a 54-year-old male with viral symptoms, found to be positive for COVID-19, who developed transient complete heart block requiring cardiopulmonary resuscitation while he was on mechanical ventilation.^11^ His rhythm recovered thereafter but he ultimately died of respiratory failure. He et al. reported a 66-year-old female who was diagnosed with COVID-19 pneumonia. At baseline, she had sinus rhythm and first-degree atrioventricular (AV) block.^12^ Following cannulation for extracorporeal membrane oxygenation (ECMO), she developed transient high-grade AV block versus complete heart block. Her rhythm thereafter returned to baseline. It is unclear whether these transient findings represent direct cardiac involvement and myocarditis, vagotonic response mediated by activated pulmonary stretch receptors in the setting of mechanical ventilation, or myocardial ischemia in the region of the conduction system.^15^

## Does COVID-19 cause myocarditis?

The concern for possible COVID-19 myocarditis masquerading as acute coronary syndrome has been raised. COVID-19 patients have been reported to present with or develop ST-segment elevation during hospitalization, with evidence of coronary and noncoronary myocardial injury.^16,17^ The mechanism of the noncoronary myocardial injury in these series is unknown. However, several case reports/series, some with ST elevation on ECG, have described possible COVID-19–associated myocarditis based on clinical presentation, positive biomarkers, conduction abnormalities, abnormalities on transthoracic echocardiogram, and/or supportive imaging **(Table 1)**.^7,18–24^ Patients who presented with ECG findings and troponin elevation concerning for acute coronary syndrome often first had significant coronary artery disease ruled out by an urgent coronary angiography or a computed tomography coronary angiogram. Case reports and online provider discussion have described the following ECG findings in patients with COVID-19: sinus tachycardia, focal ST elevation, diffuse concave ST elevation with P–R shortening, P–R prolongation, AV block, and nonspecific intraventricular conduction delay.^13,22^

Patients with COVID-19 and cardiac magnetic resonance (CMR) imaging findings consistent with myocarditis have also been described.^7,20,24^ One of these patients thereafter underwent an endomyocardial biopsy, which revealed lymphocyte-predominant infiltration and limited foci of necrosis.^7^ The SARS-CoV-2 genome was not detected on molecular analysis, leading to the final diagnosis of virus-negative lymphocytic myocarditis associated with COVID-19. Another patient also underwent an endomyocardial biopsy, which revealed viral particles in interstitial macrophages but no active inflammatory infiltration.^23^ This was thought to represent transient viremia versus the migration of infected macrophages from the lungs. Early cardiac histopathology data have otherwise been provided by autopsy reports.^25–27^

These described cases demonstrate heterogeneity in the presentation of as well as in the supporting diagnostic information obtained from patients with suspected myocarditis. For example, a clinical diagnosis of fulminant myocarditis was made in five of 68 fatal COVID-19 cases in Wuhan who deteriorated rapidly, resulting in cardiogenic shock.^28^ The heterogeneity of reported diagnostics may be affected by constraints imposed by the pandemic, including resource limitations and health care personnel exposure concerns, together with regional differences in the recommended diagnostic work-up of myocarditis in general.^29–31^

To date, autopsies have reported the absence of myocarditis during postmortem examinations of COVID-19 patients, although some authors have acknowledged that direct viral infection was unable to be ruled out. Xu et al. reported a few interstitial inflammatory cells on cardiac histopathology with no other obvious cardiac findings.^25^ Fox et al. noted scattered individual myocyte necrosis in three examined hearts. There were rare adjacent lymphocytes; however, no findings diagnostic of myocarditis were observed.^26^ Barton et al. also reported no findings of myocarditis in two examined hearts.^27^

## Electrocardiogram findings in SARS-CoV-2 infections

Alexander et al. conducted a study of rabbits infected with rabbit coronavirus.^32^ In the acute phase, they observed sinus tachycardia, reduced R-wave voltages, reduced T-wave voltages, and QT prolongation. Some rabbits were also found to have Mobitz II AV block, premature ventricular complexes, premature atrial complexes, and right bundle branch block (RBBB). Pathologic examination revealed AV nodal edema and small-to-moderate numbers of macrophages. Findings of increased lymphocytes and necrotic foci in the interventricular septum were also noted. The pathology findings of edema, degeneration, and necrosis of myocytes and the conduction system may explain the ECG findings of Mobitz II AV block and RBBB.

In humans, data on ECG changes in the setting of SARS-CoV-2 infection are still limited. In a case series of 18 patients with SARS-CoV-2 infection and ST-segment elevation, only four had diffuse ST-segment elevation, whereas the rest had focal ST-segment elevation.^16^ Of the nine patients who underwent coronary angiography, six exhibited obstructive coronary artery disease. Of the total of 18 patients, 13 died in the hospital—highlighting the poor prognosis associated with this finding in both ST-segment elevation groups. In another report of 28 COVID-19 patients with ST-segment elevation, such was reported to be focal regardless of whether or not a culprit obstructive coronary artery lesion was found on coronary angiography.^17^

## Arrhythmias in critically ill COVID-19 patients

Arrhythmia in the setting of critical illness is not uncommon and, therefore, arrhythmias in critically ill COVID-19 patients are unlikely to be specific to direct cardiac involvement. In a study of 1,341 patients admitted to the ICU with a variety of diagnoses, 12% had sustained arrhythmias.^33^ This included 6.5% with atrial fibrillation, 1.9% with atrial flutter, 1.3% with VT, and 1% with VF. Furthermore in general, respiratory infection and hypoxia are associated with arrhythmias. In a study of 32,689 patients hospitalized with pneumonia, 8% had new-onset atrial fibrillation and 1% had multifocal atrial tachycardia.^34^ In a study by Morand et al., rats exposed to intermittent hypoxia showed a higher incidence of myocardial ischemia-related ventricular arrhythmias.^35^ The possible factors associated with arrhythmias in critically ill patients with respiratory disorders include myocardial ischemia, metabolic disturbances, systemic inflammation, and elevated sympathetic tone.

## Viral myocarditis and arrhythmia

The affinity of SARS-CoV-2 for cardiac cells is under investigation. Examples of cardiotropic viruses that have been implicated in viral myocarditis include enteroviruses (eg, coxsackievirus B), adenovirus, parvovirus, and herpesviruses [eg, human herpesvirus 6 (HHV-6), Epstein–Barr virus (EBV), and cytomegalovirus], among others. Some of these viruses have certain clinical and imaging features. In mouse models of coxsackievirus B myocarditis, sinus arrest and AV block have been observed.^36^ Among humans, there have been reports made of atrial and ventricular tachycardia.^37^ Parvovirus tends to infect myocardial endothelial cells and may lead to endothelial dysfunction and an interstitial inflammatory response.^38^ On CMR imaging, late gadolinium enhancement in parvovirus myocarditis appears to localize to the epicardium of the left lateral wall.^39^ Alternatively, HHV-6 appears to localize to the anteroseptum on CMR imaging and is often intramural. In a small series of patients who presented with VT, EBV was detected by polymerase chain reaction in the endomyocardial biopsies.^40^ In the two patients with evidence of healed and nonactive myocarditis, CMR imaging showed late-gadolinium enhancement of the posterior wall. Thus, parvovirus, HHV-6, and EBV myocarditis appear to have patterns in the localization of late gadolinium enhancement on CMR imaging. Furthermore, late gadolinium enhancement itself has been shown to be an independent predictor of ventricular arrhythmia and cardiac death.^41^ Anteroseptal enhancement appears to carry the worst prognosis.^42^

## Angiotensin-converting enzyme 2 and implications for therapy

ACE2 is the functional receptor for SARS-CoV-2 entry into host cells. Besides the lungs, gastrointestinal tract, kidneys, and vasculature of these organs, ACE2 is also expressed in cardiomyocytes and is highly expressed in cardiac perivascular cells or pericytes.^2,43^ Concern has been raised regarding the continuation of ACE inhibitors and angiotensin-receptor blockers (ARBs) in patients with chronic hypertension during the pandemic. This is primarily driven by concern that these medications increase ACE2 expression, although this has only been demonstrated in animal models.^44,45^ In patients with heart failure, the use of ACE inhibitors and ARBs has not been found to increase the plasma levels of ACE2.^46^ Conversely, in animal models, ACE2 has been shown to be downregulated in the setting of infection with SARS-CoV. This downregulation of ACE2, which has anti-inflammatory effects, was thought to potentially mediate acute lung injury. Therefore, once infected with SARS-CoV-2, increased ACE2 expression could theoretically be of therapeutic benefit. In observational database- and population-based studies, an increased risk of SARS-CoV-2 infection with chronic ACE inhibitor/ARB use has not been demonstrated.^47–49^ Trials to assess the potential protective effects of ACE inhibitors, ARBs, or recombinant ACE2 are ongoing.^50^

## Hypercoagulability

Severe COVID-19 has also been associated with hypercoagulability. An elevated D-dimer level has been linked to a worse prognosis in several studies and elevated coagulation parameters such as fibrin degradation products and prothrombin time have also been correlated with worse survival.^51^ In a small series of 22 critically ill patients with COVID-19, thromboelastometry profiles were consistent with marked hypercoagulability as opposed to consumptive coagulopathy.^52^ This may be a contributory mechanism to reports of thrombosis, including an autopsy series that revealed clinically unrecognized deep venous thrombosis in seven of 12 consecutive deceased patients with COVID-19.^53^ There have also been reports of young patients presenting with SARS-CoV-2 infection and large vessel stroke and other arterial thrombi.^54,55^ It is not yet clear whether hypercoagulability in COVID-19 is provoking more instances of ST-segment–elevation myocardial infarction (STEMI). One major confounder may be decreased STEMI activation, possibly due to patient avoidance of the health care system.^56^ However, STEMI has been reported to be one presentation of COVID-19. In 28 patients with COVID-19 and ST-segment elevation on ECG, the latter was the first clinical manifestation of COVID-19 in 24 of them. Seventeen of these patients had evidence of a culprit lesion requiring revascularization, while the others did not have obstructive coronary artery disease.^17^ Prognosis was poor in both groups. The interaction of hypercoagulability, hyperinflammatory response, and pre-existing coronary artery disease in the setting of COVID-19 requires further investigation. A hyperinflammatory response with cytokine release has been observed in severe COVID-19 patients, for which preliminary studies and trials of interleukin-6–specific therapies such as tocilizumab are ongoing.^57^

## Hydroxychloroquine for the electrophysiologist

The United States Food and Drug Administration (FDA) previously issued an emergency use authorization for hydroxychloroquine and data from ongoing trials are currently pending.^50^ With regard to pharmacokinetics, hydroxychloroquine is absorbed in the upper intestinal tract. It has a long half-life of 40 to 60 days due to the large volume of distribution in the blood.^58^ In patients with renal failure, there is decreased clearance, which increases the bioavailability of hydroxychloroquine. Hydroxychloroquine is a substrate for cytochrome P450 (CYP) enzymes and, as such, it can interfere with other drugs. CYP enzymes mediate the dealkylation of hydroxychloroquine into active metabolites. These enzymes include CYP2C8, CYP3A4, CYP2D6, and CYP1A1; however, these enzymes’ contributions may vary among individuals. Relevant to cardiac electrophysiology practice, hydroxychloroquine may influence the levels of other drugs that are also metabolized by CYP2D6 by competing for the same enzyme.^58^ Therefore, plasma concentrations of drugs such as metoprolol, flecainide, and propafenone may increase.

Hydroxychloroquine blocks the delayed rectifier potassium channel I_f_ and the L-type calcium channel.^59^ Having an inhibitory effect on I_f_, it may lead to decreased heart rates. It is also possible for hydroxychloroquine to have some antifibrillatory effect based on its blockade of the potassium channel. Chloroquine has been hypothesized to be able to reduce the burden of persistent atrial fibrillation.^60^ However, chloroquine has also been associated with ventricular arrhythmias and conduction-system disorders.^61,62^ With a quinoline ring base, hydroxychloroquine has structural similarities to the class 1a antiarrhythmic drug quinidine. Therefore, there are some similarities and differences between these two drugs that are summarized in **Table 2**.

QTc prolongation has been observed in COVID-19 patients receiving hydroxychloroquine/chloroquine and torsades de pointes and VT have been reported in this setting.^63,64^ A trial administering high doses of chloroquine (600 mg twice daily) in conjunction with azithromycin in suspected cases of severe COVID-19 pneumonia was stopped due to excessive QTc prolongation and association with increased mortality.^64^ Therefore, precautionary measures are necessary to mitigate the risk of QTc prolongation.^65^ The main goal is to identify individuals who have excessive baseline QTc prolongation or those who may potentially develop an exaggerated QTc response. This includes a baseline pretreatment QTc assessment, electrolyte panel, and assessment of other medications that may prolong the QT interval or lead to significant drug–drug interactions.^65,66^ Subsequently, the decision to use exploratory SARS-CoV-2 therapy should factor in the QTc and the risk–benefit ratio. The decision on the timing of QTc monitoring following the initiation of therapy should be based on the baseline QTc.

## Conclusion

COVID-19 has several cardiovascular manifestations including conduction abnormalities, arrhythmia, and myocarditis, which are likely sequelae of cardiovascular involvement, critical illness, and/or side effects of treatments under investigation. Further characterization of SARS-CoV-2’s potential cardiac tropism and the elicited inflammatory response will inform anticipated cardiovascular disturbances and management.

## Figures and Tables

**Table 1:** Reported Cases of Suspected COVID-19–associated Myocarditis

Authors	Date of Publication*	Age, Sex	Troponin Elevation	ECG	Reduced LVEF (< 55%)	Supportive CMR***	Biopsy Obtained	Mechanical Support	Presumed Diagnosis****	Clinical Course
Sala et al.^7^	4-08-20	43, F	Yes	Mild STE with reciprocal STD	Yes	Yes	Yes		Virus-negative lymphocytic myocarditis	LVEF recovered, successfully discharged home
Zeng et al.^18^	3-11-20**	63, M	Yes	Sinus tachycardia	Yes	–	–	ECMO	Acute versus fulminant myocarditis	LVEF recovered, died secondary to sepsis
Hu et al.^19^	3-16-20	37, M	Yes	Inferior STE	Yes	–	–	–	Acute myocarditis	LVEF recovered, clinical improvement
Inciardi et al.^20^	3-27-20	53, F	Yes	Diffuse STE	Yes	Yes	–	–	Myopericarditis	Clinical improvement
Hua et al.^21^	3-30-20	47, F	Yes	Inferolateral STE	No	–	–	–	Myopericarditis complicated by tamponade	Clinical improvement after pericardiocentesis
Fried et al.^22^	4-03-20	64, F	Yes	STE	Yes	–	–	IABP	Possible myopericarditis	LVEF recovered, clinical improvement
Fried et al.^22^	4-03-20	38, M	Yes	AIVR	Yes, following arrest	–	–	ECMO	Myocarditis versus myocardial stunning versus stress cardiomyopathy	Decannulated from ECMO, still intubated at the time of report
Tavazzi et al.^23^	4-10-20	69, M	Yes	Not reported	Yes	–	Yes	IABP, ECMO	Cardiogenic shock	Cardiac function recovered, died secondary to septic shock
Kim et al.^24^	4-13-20	21, F	Yes	IVCD	Yes	Yes	–	Not reported	Myocarditis	Not reported

AIVR: accelerated idioventricular rhythm; CMR: cardiac magnetic resonance imaging; ECG: electrocardiogram; ECMO: extracorporeal membrane oxygenation; F: female; IABP: intra-aortic balloon pump; IVCD: intraventricular conduction delay; LVEF: left ventricular ejection fraction; M: male; STE: ST-segment elevation; STD: ST-segment depression.

*Date of publication format: month-day-year.

**Designates preprint publication, now published in a peer-reviewed journal.

***Refers to CMR images obtained that showed findings consistent with myocarditis.

****Refers to the diagnosis reported or the top diagnoses considered by the study authors.

**Table 2:** Comparison of Hydroxychloroquine and Quinidine

	Hydroxychloroquine	Quinidine
Half-life	40–60 days	6–8 hours
Peak plasma levels	3–4 hours	Sulfate (2 hours), gluconate (3–5 hours)
Metabolism	Hepatic metabolism to active metabolites	Hepatic metabolism to inactive compounds
Excretion	Urine (15%–25% as metabolites and unchanged drug)	Urine (5%–20% as unchanged drug)
Electrophysiologic effects, channel blockade	• Delayed rectifier potassium channel • Funny current • L-type calcium channel	• Sodium channel • Delayed rectifier potassium channel
CYP enzymes involved	Metabolized by CYP2D6 and CYP3A4	Metabolized by CYP3A4, inhibits CYP2D6

CYP: cytochrome P450.
